# The Repetitive Mechanical Tactile Stimulus Intervention Effects Depend on Input Methods

**DOI:** 10.3389/fnins.2020.00393

**Published:** 2020-04-28

**Authors:** Hiraku Watanabe, Sho Kojima, Naofumi Otsuru, Hideaki Onishi

**Affiliations:** ^1^Graduate School, Niigata University of Health and Welfare, Niigata, Japan; ^2^Institute for Human Movement and Medical Sciences, Niigata University of Health and Welfare, Niigata, Japan

**Keywords:** mechanical tactile stimulation, spatial two-point discrimination threshold, tactile threshold, Active Touch, Passive Touch

## Abstract

Elderly and stroke patients often have low spatial two-point discrimination function. The intervention effect of repetitive mechanical tactile stimulation has been shown to improve the spatial two-point discrimination function. The methods of tactile input are classified as either Active Touch or Passive Touch. In Passive Touch, the tactile stimulus is passively applied on the skin without voluntary movement, whereas in Active Touch, it is applied with voluntary movement. Based on the method of tactile input, tactile stimulation activate different cerebral cortex areas. A previous study reported that the tactile stimulation with Active Touch activate posterior parietal cortex, activated during a spatial two-point discrimination task. Therefore, the present study aimed to investigate the effects of two mechanical tactile stimulation intervention methods on two-point discrimination: tactile stimulation with voluntary movement (Active Touch) and without voluntary movement (Passive Touch). We recruited 15 healthy volunteers aged 20–23 years and applied tactile stimuli on their right index finger for 10 min. The mechanical tactile stimulator comprised 24 tiny plastic pins driven by piezoelectric actuators. In the Active Touch intervention, the pin was rubbed by voluntary movement of the right index finger (abduction 0°–10°) after the appearance of 12 pins. The Passive Touch intervention stimulated the index finger with the 12 pins setting at the centre of index finger. Tactile thresholds were measured using a two-point discrimination measurement device. Two-point discrimination threshold showed significant reduction after Active Touch intervention compared with those pre-intervention (Pre). Two-point discrimination threshold were not significantly modulated after Passive Touch intervention; however, significant negative correlation was observed between the intervention effect on two-point discrimination threshold and the performance Pre. This study suggesting that the effects of repetitive mechanical tactile stimulation depend on the method of tactile input. An effective intervention for improving two-point discrimination threshold is the application of Active Touch condition for 10 min.

## Introduction

Tactile information sensed from peripheral sensory receptors in the skin is sent to the primary somatosensory cortex (S1) via the spinal cord and the thalamus. A previous study using magnetoencephalography (MEG) recorded S1 activity corresponding to the stimulation site; it was proposed that tactile information, such as identification of a stimulation site, was processed in S1 (Nakamura et al., 1998). Higher order tactile information is processed in the secondary somatosensory cortex (S2) and posterior parietal cortex (PPC; Andersen and Buneo, 2002). The spatial two-point discrimination (2PD) is one of the indicators that reflect the higher order somatosensory function, which discriminate two spatially different two-point stimuli. 2PD is often used not only in research but also in clinical trials (Johnson and Phillips, 1981). A previous study using functional magnetic resonance (fMRI) recorded significant activity in the inferior parietal lobule (IPL) during spatial 2PD task (Akatsuka et al., 2008); it is considered that PPC including IPL is performed in higher-order sensory information processing such as spatial two-point identification.

Two-point discrimination is impaired in the elderly and stroke patients (Carey et al., 1993; Kalisch et al., 2009; Carey and Matyas, 2011; Lenz et al., 2012; Meyer et al., 2014; Franco et al., 2015; Meyer et al., 2016; Turville et al., 2019). In a previous study involving elderly patients reported that the patients who had experienced many falls had impaired 2PD of the sole compared with the ones who had experienced a few falls (Melzer et al., 2004). In a previous study involving stroke patients reported that the patients with low 2PD function also had low motor function (Meyer et al., 2016). Therefore, the 2PD and motor function of the elderly and stroke patients are closely related, and rehabilitation for improving 2PD function is considered to be important for improving somatosensory and motor functions.

The tactile stimulation is used as intervention methods for improving 2PD. A lot of previous studies have reported that repetitive mechanical tactile stimulation (rMS) improved 2PD (Godde et al., 2000; Pleger et al., 2001, 2003; Dinse et al., 2003, 2006; Hoffken et al., 2007; Kalisch et al., 2007, 2008; Ragert et al., 2008; Gatica Tossi et al., 2013; Parianen Lesemann et al., 2015; Pleger et al., 2016). For example, Pleger et al. (2003) reported that rMS applied at ∼1 Hz for 3 h on the index finger led to a decreased 2PD threshold of index finger (Pleger et al., 2003). Additionally, Ragert et al. (2008) reported that similar intervention effect was induced by applying 20 Hz for 20 min (Ragert et al., 2008). On the other hand, some previous studies question 2PD improvement after repetitive sensory input. For example, Rocchi et al. (2017) reported that repetitive sensory stimulation applied at 20 Hz for 45 min on the index finger led to a decreased somatosensory temporal discrimination threshold of the index finger, whereas spatial acuity was not improved (Rocchi et al., 2017). Additionally, Erro et al. (2018) reported that an intervention, similar to that used by Rocchi et al. (2017), in dystonia patients induced intervention effects opposite to those of healthy subjects (Erro et al., 2018). Therefore, it is necessary to consider new intervention methods because there has been no consensus on the effects of rMS intervention on 2PD.

The methods of tactile input are classified as either Active Touch or Passive Touch (Ackerley et al., 2012; Olczak et al., 2018). In Passive Touch, the tactile stimulus is passively applied on the skin without voluntary movement, whereas in Active Touch, it is applied with voluntary movement; thus, tactile, and proprioceptive information generated by movement are inputted. The mechanical tactile stimulation inputted passively induced significant S1 and S2 activities, but IPL activity was not induced (Onishi et al., 2010). On the other hand, the mechanical tactile stimulation inputted voluntarily induced significant activity of S1, S2, and IPL (Sharma et al., 2018). IPL is an important cortical area for tactile information processing by Active Touch because it is involved in sensorimotor integration (Mohan et al., 2018) and spontaneous tactile sensations (Bauer et al., 2014). For these reasons, the brain activity induced by tactile stimulation depends on methods of tactile input. Moreover, rMS with Active Touch is presumed to improve 2PD more efficiently than Passive Touch because IPL activity related to 2PD is induced by Active Touch. Therefore, the purpose of this study was to clarify the effects of different rMS intervention methods (Active and Passive Touch) on 2PD. The evaluation of 2PD may be unreliable as an index that reflects spatial acuity (Lundborg and Rosen, 2004; Tong et al., 2013). Therefore, to increase the reliability of our 2PD measurements, we used a computer with a device that precisely controlled the stimulus speed, stimulus depth, and stimulus presentation time.

## Materials and Methods

### Participants

Overall, 15 healthy volunteers [aged 20–23 years; mean ± standard division (SD): 21.0 ± 0.65 years; 13 men; 2 women] participated in this study. None of the participants reported taking any drugs or medications, which affect the central nervous system function. This study was approved by the Ethics Committee of Niigata University of Health and Welfare and was conducted in accordance with the Declaration of Helsinki. All participants provided written informed consent before participation.

### Somatosensory Function Measurement

**FIGURE 1:**
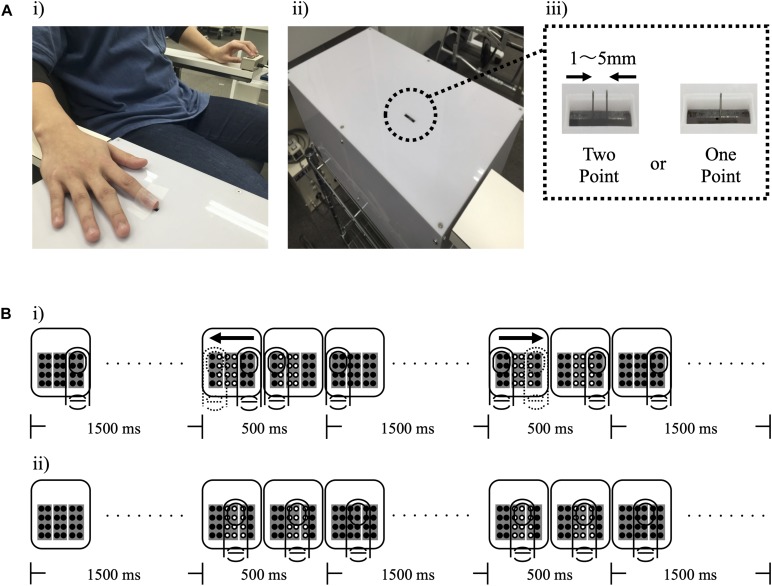
Experimental setup. **(A)** (i) shows the measurement position of study participants, (ii) shows the device for measuring the two-point discrimination threshold, and (iii) shows the two types of stimuli presented to participants. **(B)** A schematic protocol for tactile stimulation is shown. (i) and (ii) show the Active Touch and Passive Touch protocols. (○, Tactile on; ∙, Tactile off; and →, Direction of voluntary movement).

Two-point discrimination and tactile thresholds were measured as somatosensory functions. During the measurement of 2PD, subjects were comfortably seated with the right elbow in mild flexion, and forearm in a pronated position [Figure 1A(i)]. The two-point discrimination was tested using a 2PD measurement device (Takei Scientific Instruments Co., Ltd, Niigata, Japan) capable of controlling pin elevation speed, stimulation depth, and pin distance by PC [Figure 1A(ii)] (Yokota et al., 2019). Pin protrusion, elevation speed, and stimulus duration were 1 mm, 10 mm/s, and 500 ms, respectively. Overall, 10 types of tactile stimulation were used (two points of nine distances ranging from 1 mm to 5 mm in steps of 0.5 mm and one point). Each stimulation was applied 8 times, and a total of 80 stimuli were considered as 1 set. Participants were instructed to respond to the presented tactile stimulus by pressing the button as soon as possible. All ambiguous stimulus were answered as one point, whereas stimulus that reliably determined as two points were answered as two points [Figure 1A(iii)]. Tactile thresholds were also measured by monofilaments (Semmes–Weinstein monofilaments) of four varying weights (0.008, 0.02, 0.04, and 0.07 *g*). Tactile threshold was recorded using the weight that could be correctly answered three times consecutively.

### Repetitive Mechanical Tactile Stimulation

The mechanical tactile stimulator comprised 24 tiny plastic pins driven by piezoelectric actuators (TI-1101; KGS, Saitama, Japan). The dimensions of each pin were as follows: diameter, 1.3 mm; height of the protrusion, 0.8 mm; pushing force of pin, 0.031–0.12 N/pin (Onishi et al., 2010; Onishi et al., 2013; Kojima et al., 2018). The distance between pins was set at 2.4 mm. An rMS with 500 ms of protruding duration was applied to the tip of the right index finger. rMS was applied for 10 min (stim on/stim off, 0.5 s/1.5 s) under the three following conditions: Active Touch intervention, Passive Touch intervention, and Control (resting without stimulation).

In the Active Touch intervention, the pin was rubbed by active movement of the right index finger (abduction 0°–10°) after the appearance of 12 pins, and the subject was instructed to rub lightly without pushing the pin strongly [Figure 1B(i)]. The Passive Touch intervention stimulated the index finger with the 12 pins setting at the centre of index finger, and the subjects were required to count the number of stimulations for 10 min to pay attention to the tactile stimulation [Figure 1B(ii)].

### Study Design

**FIGURE 2:**
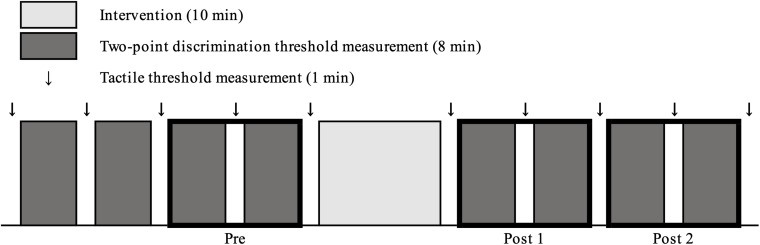
Experimental protocol. In pre-intervention measurements, four sets of two-point discrimination threshold measurements and 5 tactile threshold measurements were performed. Subsequently one of the three conditions was intervened for 10 min. After each intervention, four sets of two-point discrimination threshold measurement and 5 sets of tactile threshold measurements were performed.

For measurements pre-intervention (Pre), 4 sets of 2PD threshold measurements and 5 sets of tactile threshold measurements were performed. Thereafter, one rMS intervention or resting was performed for 10 min. After intervention, 4 sets of 2PD threshold measurements and 5 sets of tactile threshold measurements were performed. Each intervention was performed in a repeated measurement design using a randomized order, with an interval of at least 4 days between each condition (Figure 2).

### Data Analysis

For the 2PD threshold measurement data, the values from latest two sets of measurement among the total four sets before intervention were averaged and presented as values Pre. Among the four measurement sets recorded post-intervention, the average of the first two measured sets was presented as Post 1, whereas the average of the last two measured sets was presented as Post 2. The correct responses were plotted against distance as a psychometric function for absolute threshold, fitted by a logistic regression based on a generalized linear model using Matlab (Mathworks Inc.). 2PD threshold was calculated from the fit at that distance where a 50% correct response was reached (Godde et al., 2000; Pleger et al., 2001; Godde et al., 2003; Pleger et al., 2003; Hoffken et al., 2007; Ragert et al., 2008). Additionally, the just noticeable distance (JND) was calculated as an indicator of sensitivity. It was calculated from the fit at that distance where 75% and 25% correct responses were reached and was defined as the difference between the 75% and the 25% threshold (Rocchi et al., 2016). In the data obtained by using monofilaments, the results of five tactile threshold measurements pre-intervention were set as Pre 1, Pre 2, Pre 3, Pre 4, and Pre 5, whereas the results of five measurements post-intervention were Post 1, Post 2, Post 3, Post 4, and Post 5.

### Statistical Analysis

Statistical analyses were performed using SPSS statistics 25 software (IBM SPSS, Armonk, New York, NY, United States). The mean 2PD threshold were statistically analyzed by three-way repeated measures analysis of variance (ANOVA) [THRESHOLD (25% threshold, 50% threshold, and 75% threshold) × INTERVENTION (Active Touch, Passive Touch, and Control) × TIME (Pre, Post 1, and Post 2)]. The mean JND were statistically analyzed by two-way repeated measures ANOVA [INTERVENTION (Active Touch, Passive Touch, and Control) × TIME (Pre, Post 1, and Post 2)]. *Post hoc* analyses were performed using Bonferroni’s tests to compare each pre- and post- rMS intervention. Additionally, the correlation between Pre somatosensory function (2PD threshold, JND) and intervention effects were statistically analyzed by Spearman’s rank correlation coefficient. The mean tactile threshold was statistically analyzed by two-way repeated measures ANOVA [INTERVENTION (Active Touch, Passive Touch, and Control) × TIME (Pre 1, Pre 2, Pre 3, Pre 4, Pre 5, Post 1, Post 2, Post 3, Post 4, and Post 5)]. Statistical significance was set at a *P* value of <0.05.

## Results

### The Effect of rMS on 2PD Threshold

**FIGURE 3:**
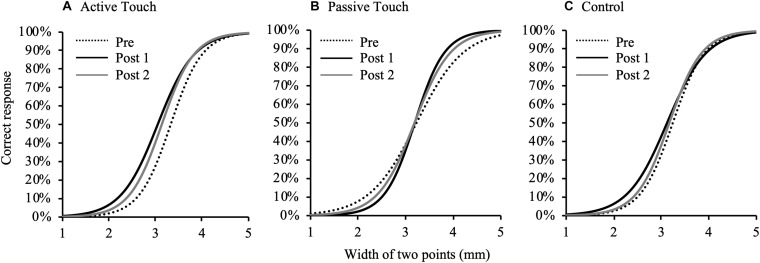
Changes in psychometric curves pre- and post- intervention under different conditions. Psychometric curves indicating plots of correct responses obtained after against width of two points of tactile stimulation and using different intervention methods. **(A)**, Active Touch; **(B)**, Passive Touch; and **(C)**, Control. Dotted line, pre-intervention measurements; black line, Post 1 measurements; gray line, and Post 2 measurements.

Figure 3 shows changes in psychometric curves before and after each intervention condition. 2PD threshold values at pre and post-intervention are presented in Table 1. Three-way repeated measures ANOVA revealed a significant effect of THRESHOLD [F_(__1_._010_,_14_._139__)_ = 210.587, *P* < 0.001, and partial eta squared (η^2^) = 0.938] and TIME [F_(__2_,_28__)_ = 6.697, *P* = 0.004, and partial η^2^ = 0.324] on 2PD threshold. In addition, there was a significant interaction between THRESHOLD, INTERVENTION, and TIME [F_(__3_._709_,_51_._920__)_ = 4.423, *P* = 0.005, and partial η^2^ = 0.240]. However, the effect of INTERVENTION [F_(__2_,_28__)_ = 0.079, *P* = 0.924, and partial η2 = 0.006] and the interaction between THRESHOLD and INTERVENTION [F_(__1_._681_,_23_._528__)_ = 0.526, *P* = 0.567, and partial η2 = 0.036], THRESHOLD and TIME [F_(__2_._034_,_28_._473__)_ = 0.542, *P* = 0.590, and partial η2 = 0.037], INTERVENTION and TIME [F_(__1_._944_,_27_._214__)_ = 1.433, *P* = 0.256, and partial η2 = 0.093] were not significant.

**TABLE 1:** 25%, 50%, and 75% threshold and just noticeable distance pre- and post-intervention in three different intervention conditions.

			**Pre**	**Post 1**	**Post 2**
Active Touch	Discrimination threshold	25% threshold	3.03 ± 0.10	2.72 ± 0.14**	2.89 ± 0.13
		50% threshold	3.33 ± 0.10	3.03 ± 0.12**	3.15 ± 0.12
		75% threshold	3.60 ± 0.09	3.35 ± 0.11**	3.42 ± 0.12*
	Just noticeable distance		0.57 ± 0.05	0.63 ± 0.07	0.53 ± 0.05
Passive Touch	Discrimination threshold	25% threshold	2.88 ± 0.19	2.93 ± 0.10	2.84 ± 0.10
		50% threshold	3.22 ± 0.17	3.19 ± 0.10	3.18 ± 0.09
		75% threshold	3.56 ± 0.16	3.45 ± 0.09	3.53 ± 0.09
	Just noticeable distance		0.68 ± 0.09	0.51 ± 0.05*	0.69 ± 0.06
Control	Discrimination threshold	25% threshold	2.95 ± 0.10	2.76 ± 0.13	2.90 ± 0.12
		50% threshold	3.25 ± 0.10	3.12 ± 0.11	3.17 ± 0.11
		75% threshold	3.53 ± 0.10	3.48 ± 0.11	3.43 ± 0.12
	Just noticeable distance		0.58 ± 0.05	0.72 ± 0.09	0.53 ± 0.05^†^

Unit; mm, *; *P* < 0.05 (vs Pre), **; *P* < 0.01 (vs Pre), †; *P* < 0.05 (vs Post 1), and (Mean ± SEM).

**FIGURE 4:**
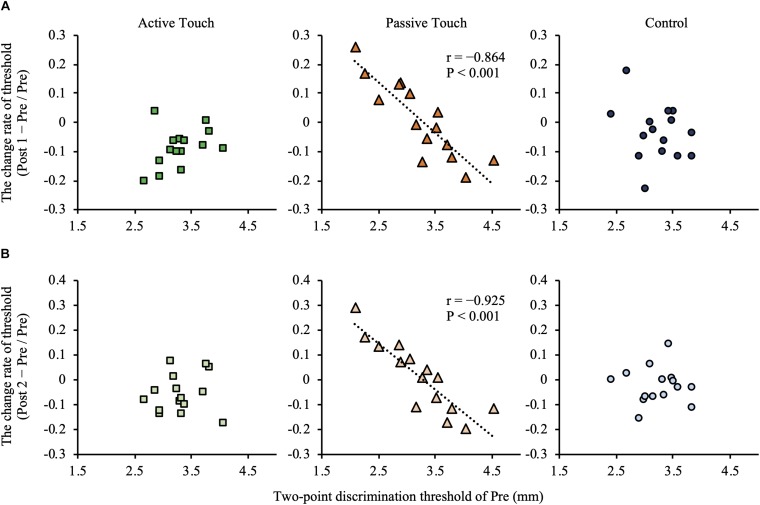
Correlation between the pre-intervention two-point discrimination threshold and intervention effect. **(A)** shows correlation between the pre-intervention two-point discrimination threshold values and intervention effect in post 1 measurements. **(B)** shows correlation between the pre-intervention two-point discrimination threshold values and intervention effect in Post 2 measurements. Under the Passive Touch condition, a negative correlation was observed between the pre-intervention two-point discrimination threshold and the change rate of two-point discrimination threshold in Post 1 [(A), middle panel] and Post 2 [(B), middle panel] (post1; *r* = −0.864, *P* < 0.001) (Post 2; *r* = −0.925, *P* < 0.001).

*Post hoc* analyses revealed that the 2PD threshold values post-intervention were significantly smaller in the Active Touch method than in Pre (25% threshold; Pre vs Post 1, *P* = 0.005) (50% threshold; Pre vs Post 1, *P* < 0.001) (75% threshold; Pre vs Post 1, *P* < 0.001, Pre vs Post 2, and *P* = 0.019). Conversely, no significant difference was observed in 2PD threshold values (*P* > 0.05) in the Passive Touch intervention and Control conditions. The Spearman’s rank correlation coefficient in Passive Touch intervention revealed a significant negative correlation between the Pre 2PD threshold and the 2PD threshold change rate after intervention (Post 1; *r* = −0.864, *P* < 0.001) (Post 2; *r* = −0.925, *P* < 0.001) (Figure 4).

### The Effect of rMS on JND

Just noticeable distance at pre- and post-intervention are presented in Table 1. Two-way repeated measures ANOVA revealed that the effect of INTERVENTION on JND [F_(__2_,_28__)_ = 0.526, *P* = 0.597, and partial η^2^ = 0.036] and TIME [F_(__2_,_28__)_ = 0.497, *P* = 0.614, and partial η^2^ = 0.034] was not significant. However, the interaction between INTERVENTION and TIME was significant [F_(__4_,_56__)_ = 4.558, *P* = 0.003, and partial η^2^ = 0.246].

**FIGURE 5:**
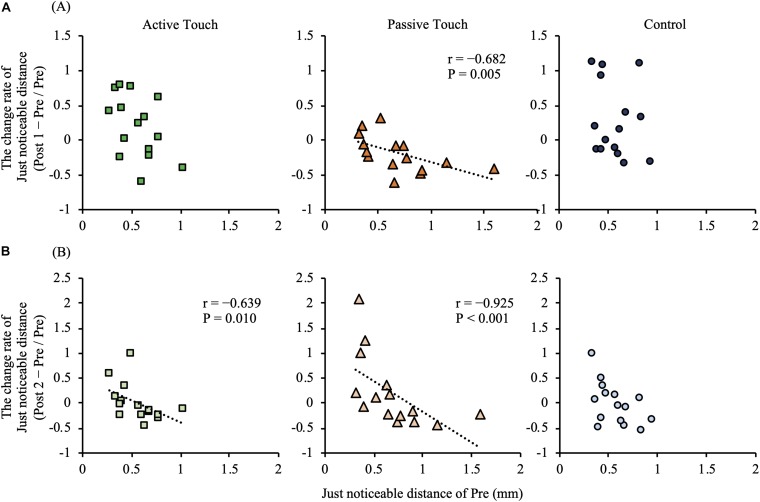
Correlation between the pre-intervention just noticeable distance and intervention effect. **(A)** shows correlation between the pre-intervention just noticeable distance and intervention effect of Post 1 measurements. **(B)** shows correlation between the pre-intervention just noticeable distance and intervention effect of Post 2 measurements. Under the Active Touch condition, a negative correlation was observed between the pre-intervention just noticeable distance and the change rate of just noticeable distance in Post 2 [(B), left panel] (Post 2: *r* = −0.639, *P* = 0.010). In the Passive Touch condition, a negative correlation was observed between the pre-intervention just noticeable distance and the change rate of just noticeable distance in Post 1 [(A), middle panel] (Post 1: *r* = −0.682, *P* = 0.005) and Post 2 [(B), middle panel] (Post 2: *r* = −0.925, *P* < 0.001).

In the Passive Touch intervention, *post hoc* analyses revealed that JND in Post 1 were significantly smaller than that in Pre (*P* = 0.030). Moreover, *post hoc* analyses revealed that JND in the Control conditions were significantly smaller in Post 2 than in Post 1 (*P* = 0.034). No significant differences between pre- and post-JND (*P* > 0.05) were observed in the Active Touch intervention. Spearman’s rank correlation coefficient revealed a significant negative correlation between JND of Pre and the change rate of JND after intervention in the Passive Touch method (Post 1; *r* = −0.682, *P* = 0.005) (Post 2; *r* = −0.925, *P* < 0.001) (Figure 5). Moreover, Spearman’s rank correlation coefficient in the Active Touch method revealed a significant negative correlation between the JND of Pre and the change rate of JND after intervention at Post 2 (Post 2; *r* = −0.639, *P* = 0.010) (Figure 5).

### The Effect of rMS on Tactile Threshold

Tactile threshold data at pre- and post-intervention are presented in Table 2. Two-way repeated measures ANOVA revealed insignificant effects of INTERVENTION [F_(__2_,_28__)_ = 0.178, *P* = 0.837, and partial η^2^ = 0.080] and TIME [F_(__9_,_126__)_ = 1.221, *P* = 0.288, and partial η^2^ = 0.013] and interaction between INTERVENTION and TIME [F_(__18_,_252__)_ = 1.165, *P* = 0.291, and partial η^2^ = 0.077].

**TABLE 2:** Tactile threshold pre- and post-intervention in three different intervention condition.

	**Pre 1**	**Pre 2**	**Pre 3**	**Pre 4**	**Pre 5**
Active Touch	0.025 ± 0.004	0.026 ± 0.004	0.026 ± 0.004	0.026 ± 0.004	0.026 ± 0.004
Passive Touch	0.024 ± 0.002	0.024 ± 0.002	0.024 ± 0.002	0.024 ± 0.002	0.024 ± 0.002
Control	0.023 ± 0.002	0.035 ± 0.012	0.023 ± 0.002	0.025 ± 0.004	0.023 ± 0.002

	**Post 1**	**Post 2**	**Post 3**	**Post 4**	**Post 5**

Active Touch	0.026 ± 0.004	0.026 ± 0.004	0.026 ± 0.004	0.026 ± 0.004	0.026 ± 0.004
Passive Touch	0.024 ± 0.002	0.024 ± 0.002	0.024 ± 0.002	0.024 ± 0.002	0.024 ± 0.002
Control	0.023 ± 0.002	0.023 ± 0.002	0.023 ± 0.002	0.023 ± 0.002	0.023 ± 0.002

*Unit; *g*, (Mean ± SEM).*

## Discussion

We investigated the effects of active and passive rMS on somatosensory function. rMS intervention of Active Touch decreased the 2PD threshold in Post 1 measurements compared with Pre. On the other hand, rMS intervention of Passive Touch led to non-significant changes, and a significant negative correlation was observed between the Pre 2PD threshold and the intervention effect. The JND was significantly improved in Post 1 compared with Pre conditions, and a significant negative correlation was observed between the Pre JND and the intervention effect. Taken together, these results suggest that the effect of rMS depends on the tactile stimulus input method.

### The Effect of rMS on 2PD Threshold

The 2PD threshold was significantly decreased in Post 1 in the Active Touch condition compared with Pre. A previous study using fMRI reported that IPL activity are involved in the integration of proprioceptive sensory information and tactile information (Naito and Ehrsson, 2006). In the Active Touch condition, tactile information were inputted with proprioceptive information of finger movements. Therefore, it was presumed that the IPL was repeatedly activated during Active Touch intervention. Moreover, a previous study using fMRI reported that the activation of IPL is induced by tactile stimulus moving on the finger pad (Wacker et al., 2011). The IPL was activated during the spatial integration of information from one site to another; the activity of the left IPL was specifically recognized during the 2PD task (Akatsuka et al., 2008). For these reasons, the decreased 2PD threshold observed in Active Touch condition may reflect the changing activity of the left IPL. On the other hand, several studies have also reported that the modulation of 2PD threshold is associated with changes in S1 activity. For example, Pleger et al. (2003) reported that a positive correlation was observed between increasing S1 activity and decreasing 2PD threshold following rMS intervention (Pleger et al., 2003), Additionally, Hoffken et al. (2007) reported that a positive correlation was observed between reduced S1 inhibitory activity and 2PD threshold reduction following rMS intervention (Hoffken et al., 2007). Therefore, there is a possibility that the modulation of 2PD threshold was caused by the change in S1 activity after Active Touch intervention. However, the intervention effect of Active Touch is thought to be greatly influenced by the activity change in the cerebral cortex area that is responsible for higher somatosensory processing because no change in the 2PD threshold was observed after rMS intervention with Passive Touch.

The 2PD threshold did not show any statistically significant changes in the Passive Touch condition, whereas a significant negative correlation was observed between the intervention effect and Pre 2PD threshold. This result suggests that the effects of Passive Touch intervention on 2PD threshold depend on the threshold in Pre. In previous studies, the tactile stimulus with stimulation frequency of 1 Hz applied for 3 h decreased the 2PD threshold (Pleger et al., 2003), whereas the tactile stimulus with 1 Hz for 20 min increased the 2PD threshold and that with 20 Hz for 20 min decreased the 2PD threshold (Ragert et al., 2008). Thus, intervention effect of rMS clearly depends on stimulation frequency and intervention method (Godde et al., 2000; Pleger et al., 2001; Godde et al., 2003; Ragert et al., 2008; Parianen Lesemann et al., 2015). We applied half of stimulation frequency/duration (0.5 Hz for 10 min) reported in a previous study (1 Hz for 20 min; Ragert et al., 2008); therefore, 2PD threshold was not changed by Passive Touch intervention. On the other hand, a significant negative correlation was observed between the intervention effect and Pre 2PD threshold. A previous study that measured the grating orientation discrimination threshold before and immediately after peripheral electrical stimulus intervention reported that the intervention effect depended on the before intervention threshold (Saito et al., 2018), which is consistent with the results obtained in our study. This intervention effect is related to changes in paired pulse depression (PPD), which is an indicator of inhibitory activity of primary somatosensory cortex. Therefore, the intervention effect of the Passive Touch condition in this study may involve changes in the inhibitory activity of the primary somatosensory cortex.

The evaluation of 2PD may be unreliable as an index that reflects spatial acuity (Lundborg and Rosen, 2004; Tong et al., 2013). Consequently, several researchers have avoided using 2PD to measure spatial acuity due to its unreliability (Rocchi et al., 2016; Rocchi et al., 2017; Erro et al., 2018). This low reliability may be based on the fluctuation of the stimulus presentation area, presentation speed, and/or presentation pressure. Therefore, to increase the reliability of our 2PD measurements, we used a computer with a device that precisely controlled the stimulus speed, stimulus depth, and stimulus presentation time. Moreover, the stimulus settings used for evaluation were based on a previous study, in which stable results were obtained with multiple measurements (Yokota et al., 2019). Therefore, we consider that the changes in 2PD threshold measured in this study accurately reflect the changes in spatial acuity.

### The Effect of rMS on JND

JND was significantly decreased in Post 1 in the Passive Touch condition compared with Pre. In this study, the change of the 50% threshold is not observed after Passive Touch intervention, whereas the number of subjects with the 25% threshold approaching the 50% threshold was 7/15, and the number of subjects with the 75% threshold approaching the 50% threshold was 8/15. The JND was calculated from the 25% threshold and the 75% threshold, therefore it was considered that either change was reflected. In this study, the number of subjects with 25% threshold or 75% threshold approached 50% threshold was 14/15, and it was observed the statistically significant difference of JND following these modulations.

### Significance of Clinical Sites

The intervention effect of rMS with Active Touch in this study was recognized after 10 min. Pumpa et al. (2015) had reported that 72% of therapists complain of lack of time for rehabilitation aimed at improving somatosensory function (Pumpa et al., 2015). The shortest stimulation time for rMS that showed intervention effects in previous studies was 20 min; The 10 min intervention time in this study is the shortest intervention in the currently reported research on rMS. Therefore, rMS with Active Touch in this study is an intervention method that can efficiently improve somatosensory function, suggesting that it may be an effective intervention method in situations when rehabilitation time is restricted. However, this study is for healthy adults, and it is unclear whether it can be applied to patients with reduced somatosensory functions. The intervention of repetitive sensory stimulation for healthy subjects improved somatosensory function (Rocchi et al., 2017); however, when this stimulation was applied to dystonia patients, it induced intervention effects that were opposite to those of healthy subjects (Erro et al., 2018). Therefore, the intervention effects on patients with impaired sensory function require further investigation.

### Study Limitations

The limitation of this study was that not cortical activity such as IPL or S1 and PPD were not measured. Multiple cerebral cortical regions, such as the S2, PPC, insula cortex, and premotor cortex, are involved in processing tactile input (Dijkerman and de Haan, 2007; Martuzzi et al., 2014; Rullmann et al., 2019). Moreover, subcortical regions such as the putamen and cerebellum are involved in processing tactile input with movement (Merchant et al., 1997; van der Zwaag et al., 2013). Therefore, these cerebral cortical and subcortical regions may be related to the results reported in the present study; however, the data from our experiment cannot confirm this hypothesis. Further investigation is therefore required into the changes in cortical and subcortical activities after Active and Passive Touch intervention.

## Conclusion

We investigated the effect of rMS intervention with Active Touch and Passive Touch on spatial two-point discrimination. The effect of rMS intervention depends on the tactile stimulus input method. An effective intervention for improving 2PD threshold is the application of Active Touch condition for 10 min.

## Data Availability Statement

The datasets generated for this study are available on request to the corresponding author.

## Ethics Statement

The studies involving human participants were reviewed and approved by Niigata University of Health and Welfare. The patients/participants provided their written informed consent to participate in this study.

## Author Contributions

HW, SK designed the experiment, recorded and analyzed data, and wrote this manuscript. NO and HO designed the experiment, conducted the statistical analysis, and edited and revised this manuscript.

## Conflict of Interest

The authors declare that the research was conducted in the absence of any commercial or financial relationships that could be construed as a potential conflict of interest.
